# Histone Epigenetic Signatures in Embryonic Limb Interdigital Cells Fated to Die

**DOI:** 10.3390/cells10040911

**Published:** 2021-04-15

**Authors:** Cristina Sanchez-Fernandez, Carlos I. Lorda-Diez, Cristina Duarte-Olivenza, Juan M. Hurle, Juan A. Montero

**Affiliations:** Departamento de Anatomía y Biología Celular, Instituto de Investigación Sanitaria Marqués de Valdecilla (IDIVAL), Universidad de Cantabria, 39011 Santander, Spain; Lordaci@unican.es (C.I.L.-D.); cristina.duarte@alumnos.unican.es (C.D.-O.); hurlej@unican.es (J.M.H.)

**Keywords:** apoptosis, programmed cell death, trichostatin A, FGF8, BMP2, BMP4, BMP5, BMP7, developmental cell senescence, histone deacetylases

## Abstract

During limb formation in vertebrates with free digits, the interdigital mesoderm is eliminated by a massive degeneration process that involves apoptosis and cell senescence. The degradation process is preceded by intense DNA damage in zones located close to methylated DNA, accompanied by the activation of the DNA repair response. In this study, we show that trimethylated histone 3 (H3K4me3, H3K9me3, and H3K27me3) overlaps with zones positive for 5mC in the nuclei of interdigital cells. This pattern contrasts with the widespread distribution of acetylated histones (H3K9ac and H4ac) and the histone variant H3.3 throughout the nucleoplasm. Consistent with the intense labeling of acetylated histones, the histone deacetylase genes *Hdac1*, *Hdac2*, *Hdac3*, and *Hdac8*, and at a more reduced level, *Hdac10*, are expressed in the interdigits. Furthermore, local treatments with the histone deacetylase inhibitor trichostatin A, which promotes an open chromatin state, induces massive cell death and transcriptional changes reminiscent of, but preceding, the physiological process of interdigit remodeling. Together, these findings suggest that the epigenetic profile of the interdigital mesoderm contributes to the sensitivity to DNA damage that precedes apoptosis during tissue regression.

## 1. Introduction

Digit primordia are the last skeletal components of the limb to be formed during development. During their formation, the skeletal progenitors of the autopod segment of the limb aggregate in radial condensations separated by interdigital regions. This process is locally regulated by growth factors, such as TGF-β or BMPs, and in species with free digits, involves massive elimination of interdigital cells [1,2,3,4]. For many years, interdigit remodeling was described as an altruistic apoptotic “cellular suicide” process; currently, it is well known as a complex process involving several degenerative pathways, including apoptosis mediated by caspases, cell death associated with lysosomal activation, and cell senescence [5,6,7,8]. In addition to these degradative routes, oxidative stress might exert an important role in the establishment of areas of interdigital cell death [9,10,11]. Although we do not know yet how these degenerative routes are coordinated or whether they are regulated by other signals, it has been shown that interdigit degradation is preceded by intense DNA damage and by activation of a DNA repair response [12,13]. A similar process also occurs in cancer and is associated with epigenetic alterations, such as DNA methylation or post-translational histone modifications [14]. We have recently found that prior to the onset of cell death, interdigital progenitors bear increased genome instability compared to the digit-forming precursors. This difference was associated with changes in global and regional DNA methylation mediated by UHRFs (ubiquitin-like containing plant homeodomain and RING finger domain) and DNMTs (DNA methyltransferases) [15,16].

As occurs with DNA methylation, histone post-translational modifications (PTMs) can affect gene expression by recruiting histone modifiers and altering the chromatin structure [17]. Histone methylation and acetylation are covalent modifications of histone proteins mediated by enzymatic activity [18]. The transfer of methyl groups to lysine or arginine residues on the histone side chains is mediated by histone methyltransferases (HMTs) and histone demethylases (HDMs) [19]. Histone methylation is usually associated with transcriptional repression or gene silencing [20,21]. In contrast, histone acetyltransferases (HATs) and histone deacetylases (HDACs) regulate histone acetylation on lysine residues, resulting in activation of transcriptional effects [22]. Thus, the addition of the acyl group reduces the affinity of the histone tail for chromatin, leaving the underlying DNA more exposed and exposing binding sites for proteins bearing a bromodomain with a pro-transcriptional function [23]. Histone acetylation is involved in the regulation of many cellular processes, including chromatin dynamics and transcription, cell cycle progression, differentiation, DNA replication, and DNA repair [24]. The implication of histone post-translational modifications mediated by HDACs has been described in some degenerative processes [25,26,27]. However, inhibition of histone deacetylases can attenuate pro-apoptotic stimuli in some animal models of neurodegeneration [28,29].

In the developing limb, the acquisition of distinctive epigenetic signatures has been associated with various events, including proximodistal specification, myogenic differentiation, endochondral ossification, or articular cartilage formation [30,31,32,33,34]. The purpose of this study was to initiate characterization of the pattern of histone methylation and acetylation of interdigital progenitors during the remodeling of interdigital tissue to uncover their possible implications in this degenerative process.

## 2. Materials and Methods

We employed Rhode Island chicken embryos from 5 to 8 days of incubation (id), which is equivalent to stages 25 to 33 HH. In all cases experiments were done in the third interdigit of the right leg bud.

### 2.1. Neutral Red Staining, TUNEL Assay, and Histochemical Detection of β-Gal Activity

Neutral red is a vital stain used for the detection of cell death and/or cell senescence [8]. For this purpose, autopods were dissected free and immersed in 0.01% neutral red diluted in PBS at 37 °C. In some cases, the specimens were washed in PBS after staining, fixed in neutral formol-calcium, and sectioned in the vibratome and examined under the binocular microscope.

Detection of β-galactosidase activity was performed at pH 6 in vibratome sections of specimens fixed in 4% glutaraldehyde.

Apoptotic DNA fragmentation was detected using the TUNEL assay in vibratome tissue sections and the in situ cell death detection kit (Roche Diagnostics GmbH, Manheim, Germany) according to the manufacturer’s instructions.

### 2.2. Immunofluorescence and Confocal Microscopy

For immunolabeling studies, we employed limb tissue samples fixed in 4% PFA, squashed interdigital tissue fragments, or vibratome sections permeabilized with Triton X-100 in PBS. We used the following antibodies (1:100): mouse monoclonal anti 5-methylcytosine (5mC; Eurogentec, Searing, Belgium), rabbit polyclonal anti-H3 lysine 4 trimethylated (H3K4me3; NB-21-1023, Novus Biologicals, Abingdon, UK), rabbit polyclonal anti-H3 lysine 9 trimethylated (H3K9me3; ab8898, Abcam, Cambridge, UK), rabbit polyclonal anti-H3 lysine 27 trimethylated (H3K27me3; A2363, ABclonal, Woburn, MA, USA), rabbit polyclonal anti-H3 lysine 9 acetylation (H3K9ac; 06-943, Millipore-Upstate), rabbit polyclonal anti-histone H3.3 (NBP2-24697, Novus Biologicals, Abingdon, UK), rabbit polyclonal anti-H4 acetylated (H4ac; 06-598, Millipore-Upstate, Temecula, CA, USA), and mouse monoclonal anti-phospho-histone H2AX (Ser 139; JBW301 Millipore-Upstate, Temecula, CA, USA). These antibodies were incubated with specific secondary antibodies conjugated with FITC or Texas Red (1:75; Jackson).

Counterstaining to distinguish nuclei was performed using DAPI (Vector Laboratories), with observations using a LSM510 Laser Confocal Microscope (Zeiss).

For quantification experiments, the number of histone PTM foci (H3K4m3, H3K9m3, and H3K27m3) that overlapped with DNA methylation regions positive for 5mC immunolabeling was assessed in squash preparations and was estimated by direct examination using a 63× objective. The nuclear area was determined using DAPI staining and was measured with ImageJ default tools. First, the nuclear area was determined and then the regions occupied by 5mC were delimited by applying an automatic local threshold. 

### 2.3. Experimental Manipulation of Interdigit Regression

To study the effect of trichostatin A (Sigma, Munich, Germany), a histone deacetylase inhibitor, we implanted AG1X-2 (BioRad, Hercules, CA, USA) beads incubated in 750 µM TSA in the third interdigit of the leg bud at id 5.5. The implication of BMP signaling in the effects of TSA treatments was analyzed by implanting a heparin bead (Sigma, Munich, Germany) incubated in 1 mg/mL rh-Noggin (Preprotech, London, UK) together with the TSA bead. The influence of FGF signaling was analyzed by implanting a heparin bead incubated in 0.5 mg/mL FGF2 (R&D Systems, Minneapolis, MN, USA) together with the TSA bead. Treatments with beads soaked in PBS or DMSO were employed as controls in all experiments. 

### 2.4. In Situ Hybridization

*Hdac1*, *Hdac2*, *Hdac3*, *Hdac8*, and *Hdac10* RNA probes were obtained by PCR from chick limb buds at initial stages of digit formation. Specific primers for chick *Hdac1* were 5′-ttcaccacgctaagaagtcg-3′ and 5′-cacgttgcggatcgtatagc-3′; for chick *Hdac2*, 5′-ttgccattgctgatgttagg-3′ and 5′-ttcaccactgttgtccttgg-3′; for chick *Hdac3*: 5′-gtgtttccagggctctttga-3′ and 5′-acccagagaatctgcaccac-3′; for chick *Hdac8*, 5′-ggcatcacgcaaagaaagat-3′ and 5′-tcctcccaggatgagagttg-3′; and for chick *Hdac10*: 5′-aacaacagctgagttggaagc-3′ and 5′-ccgtgttcggaatgatatgc-3′.

In situ hybridization of PFA-fixed limbs samples was performed in whole mounted or 150 µm vibratome sections. After proteinase K (10 µg/mL) treatments for 20–30 min at 20 °C, hybridization with digoxigenin-labeled antisense RNA probes was performed at 68 °C and alkaline phosphatase-conjugated anti-digoxigenin antibody (1:2000; Roche). Reactions were developed with BM purple AP substrate precipitation (Roche).

### 2.5. Real Time Quantitative PCR (qPCR) for Gene Expression Analysis

The expression levels of *Fgf8* and interdigital expressed *Bmp* genes were analyzed by qPCR in control interdigits and TSA-treated interdigits 7 h after bead implantation. Total RNA was extracted from interdigital tissue samples consisting of pools of 12 interdigits (see Figure 6A). Total RNA concentration and its purity were assessed using a Nanodrop spectrophotometer (ND-1000). First-strand cDNA was synthesized employing the High Capacity cDNA Reverse Transcription Kit (Life Technologies Carlsbad, CA, USA). The cDNA concentration measured in a Nanodrop spectrophotometer (ND-1000) was adjusted to 0.5 μg/μL. qPCR analysis was performed using the Mx3005P system (Stratagene, San Diego, CA, USA) with automation attachment. In this work, we have used SYBRGreen (Life Technologies)-based qPCR and GAPDH was chosen as the normalizer gene. A total of four control and four TSA-treated samples were analyzed. Mean values for gene expression fold changes were measured and evaluated relative to a calibrator according to the 2^−ΔΔCt^ equation [35]. Student’s T test for statistical comparison were done using SPSS for Windows v.18.0, and the statistical significance was set at *p* < 0.05. Specific oligos for chick genes were as follows: for *Fgf8*, 5′-cgtgttcatgcacttgttcg-3′ and 5′-gatctgtcaccaggctctgc-3′; for *Bmp2*, 5′-tggaatgactggattgttgc-3′ and 5′-tggaattcaccgaattgacc-3′; for *Bmp4*, 5′-agagcctccaggagatcagc-3′ and 5′-gctgaggttgaagacgaagc-3′; for *Bmp5*, 5′-cagcgaaggcactacaagg-3′ and 5′-gctgctgtcactgcttctcc-3′; and for *Bmp7*, 5′-aagcacgagctctatgtcagc-3′ and 5′-cacagtaatacgcagcatagcc-3′.

## 3. Results

### 3.1. Post-Translational Histone Modifications (PTMs) of Interdigital Progenitors Fated to Die

We previously observed by, immunohistochemistry, that before interdigit regression, the nuclei of interdigital cells show γH2AX-positive DNA breaks associated with 5-methylcytosine (5mC) foci, representing a pattern of DNA methylation [16]. Here, we have monitored the pattern of histone methylation and acetylation in dissociated interdigital cells and their relationship with methylated DNA. For this purpose, we selected H3 lysine 4 trimethylation (H3K4me3), H3 lysine 9 trimethylation (H3K9me3), H3 lysine 27 trimethylation (H3K27me3), H3 lysine 9 acetylation (H3K9ac), the histone variant H3.3, and H4 acetylation (H4ac) for immunolabeling. 

**Figure 1:**
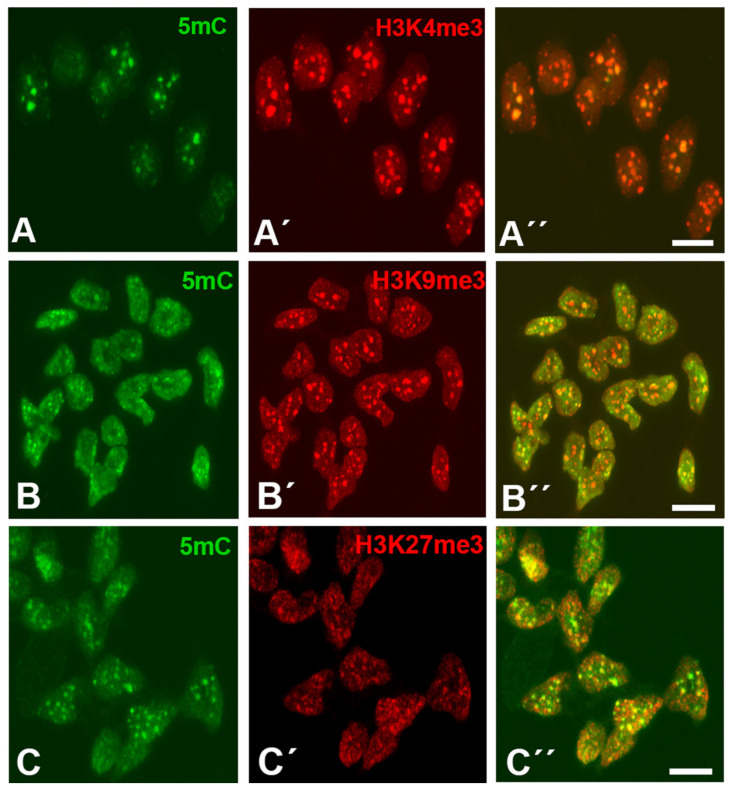
Immunolabeling of H3K4me3(**A’**,**A”**), H3K9me3 (**B’**,**B”**), and H3K27me3 (**C’**,**C”**) (red labeling) in combination with 5mC (green labeling, **A**,**B**,**C**) in dissociated cells from the third interdigits of leg buds at id 6 (stage 29 HH). Note in the merged images (**A”**,**B”**,**C”**) that all three trimethylated histones show an dotted expression pattern overlapping with foci positive for 5mC (**A”**,**B”**,**C”**). Magnification bars = 5 µm.

As shown in Figure 1A–C, interdigital cells showed robust H3K4me3, H3K9me3, and H3K27me3 labeling in a characteristic dotted pattern that broadly overlapped with 5mC marks. The H3K4me^3^ and H3K9me3 marks showed variable sizes ranging from dots of 0.2 µm in diameter up to clumps of 0.4 µm, while H3K27me3 dots exhibited a smaller size (between 0.04 and 0.10 µm). As shown in Figure 1, these marks were associated with 5mC foci (Figure 1A–C). 

After analyzing the distribution of a total of 650 H3K4me3, H3K9me3, and H3K27me3 foci from 10 different samples of interdigit tissue at id 6 (stage 29 HH), we have observed that 494 (76%), 409 (63%), and 357 (55%) of the foci overlapped with DNA methylation regions positive for 5mC immunolabeling. This association is more remarkable considering that 5mC foci occupied approximately 10% of the total surface of the nuclei. In all cases, during the course of degeneration, the immunolabeled foci tend to be reduced in number, consistent with the intense chromatin alterations and DNA fragmentation that take place during apoptotic cell death [36,37]. 

**Figure 2:**
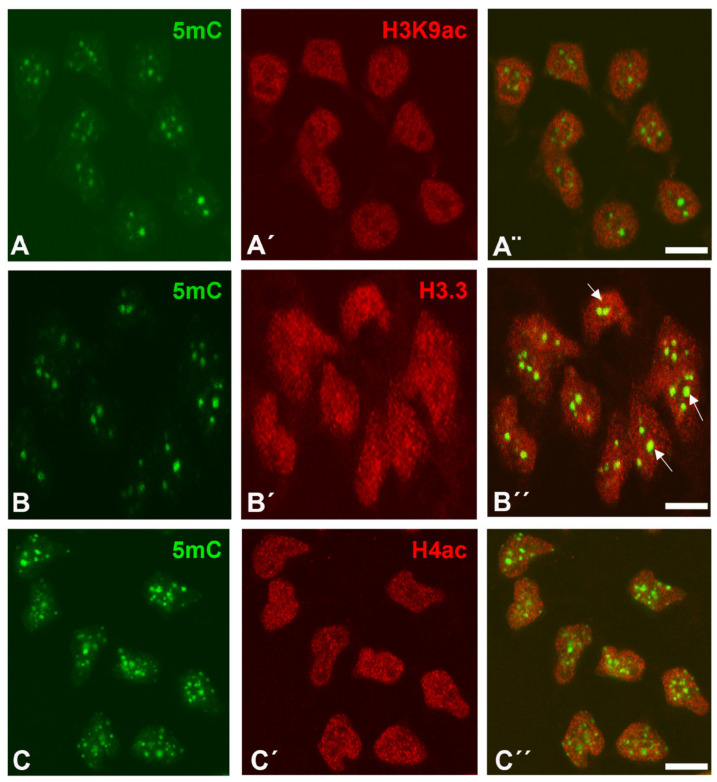
Immunolabeling of H3K9ac (**A’**,**A”**), H3.3 (**B’**,**B”**), and H4ac (**C’**,**C”**) (red labeling), in combination with 5mC (green labeling, **A**,**B**,**C**) in dissociated cells from the third interdigits of leg buds at id 6 (stage 29 HH). Note that H3K9ac (**A**–**A”**) and H4ac (**C**–**C”**) show a diffuse nuclear distribution that does not occupy zones of DNA methylation positive for 5mC immunolabeling (**A**–**C**). Differently from H3K9ac and H4ac, H3.3 (**B’**,**B”**) also shows diffuse labeling within the nucleoplasm but overlapping in some zones (arrows in **B”**) with 5mC marks. (**A”**,**B”**,**C”**) are merged images. Magnification bars = 5 µm.

With respect to difference in the trimethylated histones, H3K9ac, H3.3, and H4ac showed a widespread punctate distribution within the nuclei of the interdigital progenitors. The positive foci were smaller (ranging between 0.05–0.20 µm); H3K9ac and H4ac immunolabelling appeared predominantly distributed in 5mC-negative areas of the nuclei (Figure 2A–C). This localization differs from that of trimethylated histones and is consistent with the inverse relationship between acetylation and methylation marks reported by numerous studies [38,39]. Distinct to the acetylated histones, a reduced number of H3.3-positive foci overlapped with 5mC marks.

### 3.2. Expression Domains of HDACs in the Embryonic Limb

**Figure 3:**
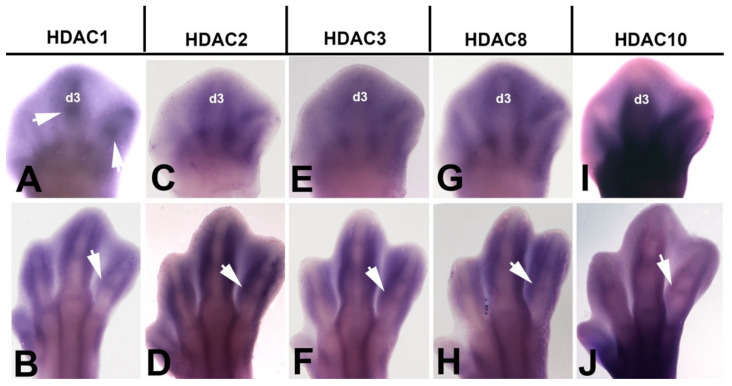
In situ hybridizations showing the interdigital expression of HDAC gene family at id 5.5 (28HH) (**A**,**C**,**E**,**G**,**I**) and 7.5 (32HH) (**B**,**D**,**F**,**H**,**J**). Note that class I HDAC genes, including *Hdac1*, *Hdac2*, *Hdac3*, and *Hdac8*, are expressed in the interdigital mesoderm and also in the joint regions. The class II gene *Hdac10* (**I**,**J**) is expressed at lower levels than class I genes, but joint domains (arrow) are still identified at id 7.5 (**J**). Arrows indicate the expression domains in the developing interphalangeal joints. Digit 3 is indicated in all id 5.5 limbs as d3. Bar = 500 µm.

Histone acetylation on lysine residues is tightly regulated by opposing actions of two families of enzymes, histone acetyltransferases (HATs) and histone deacetylases (HDACs) [40]. Hyperacetylation of histone tails induced by HATs results in an open chromatin formation that usually correlates with gene activation, whereas deacetylation by HDACs mediate a closed chromatin conformation and transcriptional suppression [41]. To further explore the presence of post-translational histone modifications in the regressing interdigits, we analyzed the expression of HDACs genes in the developing autopod by in situ hybridization (Figure 3). We detected intense autopodial expression of class 1 HDAC members *Hdac1*, *Hdac2*, *Hdac3*, and *Hdac8*. Expression domains of these genes included the interdigital spaces at id 5.5 and 7.5 and the developing joints (Figure 3A–H) that are areas where cells undergo programmed cell death and senescence. In addition to the class1 HDAC members, *Hdac10*, that belong to the class IIb HDAC family was also expressed in the autopod, but at considerably lower levels (Figure 3I,J). Differently to the characteristic nuclear localization of class 1 HDAC members, Hdac3 and class IIb members also show cytoplasmic localization [42,43].

### 3.3. Inhibition of Histone Deacetylase and Cell Death

**Figure 4:**
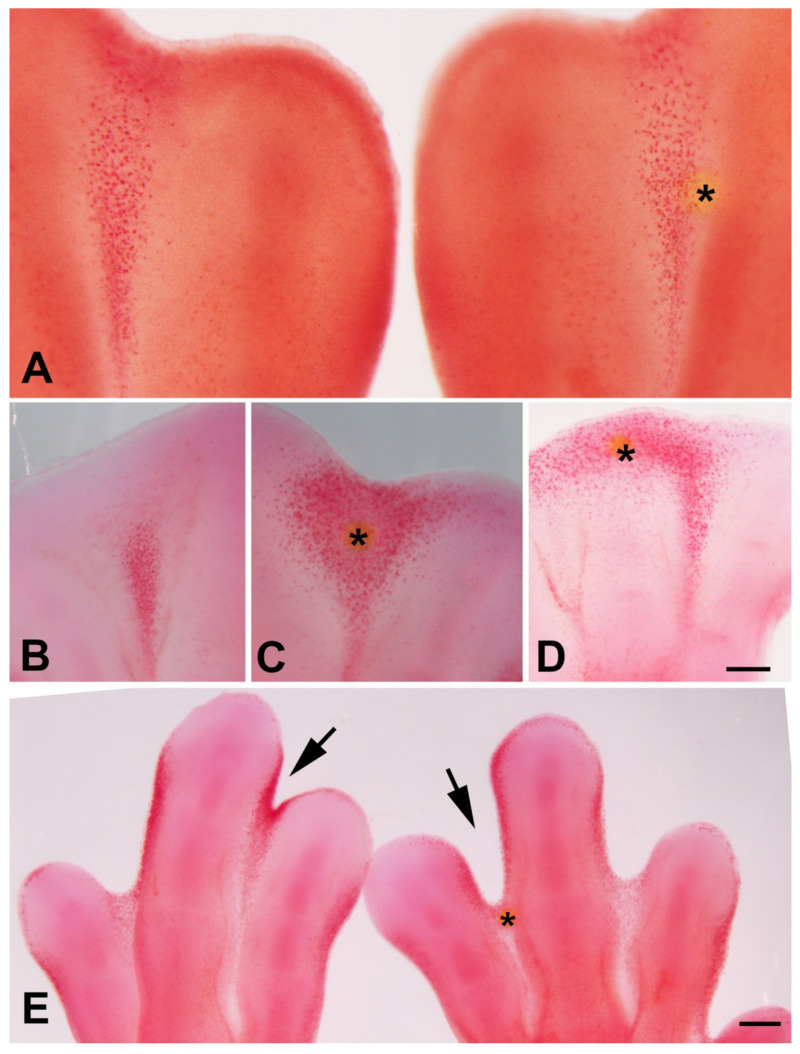
TSA induces cell death and DNA damage. (**A**) Interdigital spaces neutral red vital stained 36 h after the implantation of a PBS bead (*) in the right limb. Note that the pattern of interdigital cell death has not been changed in the interdigits subjected to implantation of a control bead. (**C**,**D**) Control (**left**) (**C**) and experimental (right) interdigits (**D**) vital stained with neutral red to illustrate the intense induction of cell death 24 h after implantation of a TSA bead (*). (**D**) Experimental autopod showing the pattern of cell death induced by implantation of a TSA bead at the tip of the growing digit III. Note that death extends through the undifferentiated mesoderm while it is absent at the cartilaginous end of the digit close to the bead (*). (**E**) Control (left) and experimental (right) autopod vital stained with neutral red 48 h after implantation of a TSA bead (*). Note the advanced stage on interdigital remodeling in the treat interdigits in comparison with its control right autopod (arrows). Magnification bar in (**A**–**C**) = 200 µm; bar in (**D**) = 300 µm.

Trichostatin A (TSA) is a potent and noncompetitive reversible inhibitor of type I and type II HDACs that induces growth arrest, cell differentiation, and apoptosis in tumor cells [44,45,46]. Previous studies have observed that local application of trichostatin A to early limb bud promoted cell death in the mesenchymal core of the bud accompanied by transcriptional regulation of genes responsible for myogenic differentiation and limb patterning [30,32]. The expression of *Hdac* genes in the interdigits and in the developing interphalangeal joints, that are regions where programmed cell death occurs, prompting us to explore the effects of local inhibition of histone deacetylases by implanting beads bearing trichostatin A in the stages preceding cell death (Figure 3) [13]. Control beads incubated in PBS only did not change the pattern of interdigital tissue degeneration (Figure 4A). 

**Figure 5:**
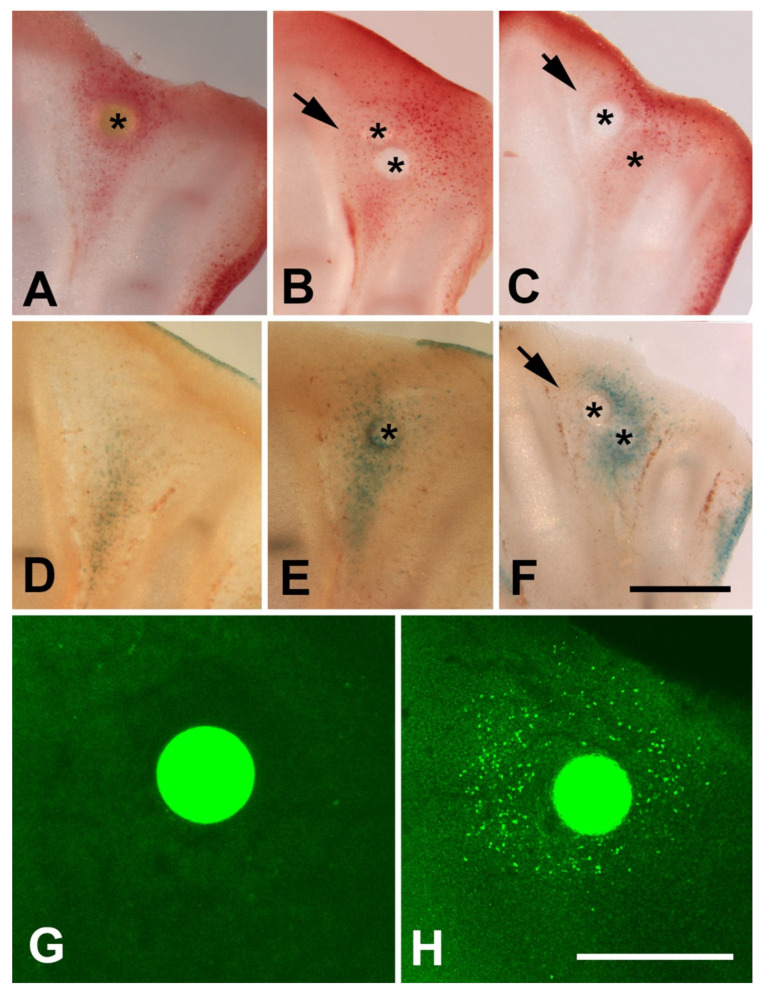
(**A**–**C**) Vibratome sections of autopods 24 h after interdigital implantation of a TSA bead alone (**A**), double implantation of Noggin and TSA bead (**B**), and double implantation of FGF bead and TSA bead (**C**). (**D**–**F**) Vibratome sections showing the pattern of β-galactosidase activity in control untreated (**D**) 24 h after implantation of a TSA bead, and 24 h after double implantation of a FGF bead and a TSA bead. Asterisks indicate the position of the beads. Note the inhibition of neutral red labeling and β-galactosidase activity in the zone close to Noggin and FGF bead (arrows) in comparison with single TSA treatments in (**A**,**E**). (**G**,**H**) Absence of γH2AX-positive cells 8 h after implantation of a control PBS bead (**G**) and intense labeling after implantation of a TSA bead (**H**). Magnification bar in (**A**–**F**) = 400 µm; bars in (**G**,**H**) = 200 µm. Asterisks in images (**A**–**F**) show the position of the beads.

Twenty-four hours after TSA bead implantation, massive cell death and cell senescence were induced around the bead, including the apical ectodermal ridge (AER) of the interdigital region (*n* = 12; Figure 4B,C and Figure 5D,E). When the beads were implanted at the tip of the digits (*n* = 6), cell death was induced in the undifferentiated progenitors located distally to the digit tip, but cell death was almost absent proximally to the bead in the region of cartilage differentiation (Figure 4D). At 48 h after the treatment, interdigits appeared to be in an advanced stage of regression compared to the contralateral control limb (*n* = 5; Figure 4E). In contrast, treatments applied at the tip of the digits (*n* = 6) abrogated digit outgrowth. 

**Figure 6:**
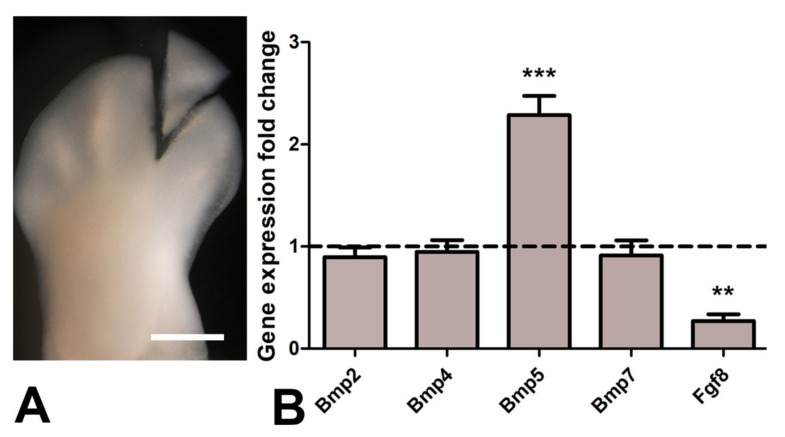
(**A**), representative image of the autopod after microsurgery to dissect free the interdigital tissues employed for qPCR analysis and immunocytochemistry. Magnification bar = 300 µm. (**B**) qPCR comparative analysis of interdigital expressed *Bmp* genes (*Bmp2*, *Bmp4*, *Bmp5*, *Bmp7*) and *Fgf8* in control interdigits and in experimental interdigits 7 h after the implantation of a TSA bead. The dotted line represents the expression level of each gene in the control samples. Note upregulation of *Bmp5* and intense downregulation of *Fgf8* in the treated interdigits. ** *p* < 0.01; *** *p* < 0.001.

It is well known that physiological interdigital cell death is inhibited by local application of BMP antagonists, such as Noggin, and by local application of FGF2 [47]. To determine whether cell degeneration induced by TSA was comparable to that happening physiologically, we performed experiments of double implantation of a Noggin bead together with a TSA bead (*n* = 8) and double implantation of a FGF2 bead together with a TSA bead (*n* = 7). Degeneration was analyzed by histochemical detection of β-gal activity, TUNEL, and neutral red vital staining. Both Noggin and FGF beads reduced cell death and cell senescence associated with the TSA treatment alone (Figure 5A–F), but the intensity of inhibition was more accentuated after FGF bead treatment. Consistent with these differences in cell death intensity, transcriptional analysis of interdigits 7 h after bead application showed intense downregulation of Fgf8 gene and upregulation of Bmp5, but not Bmp4, Bmp2, nor Bmp7 (Figure 6). FGF8 is a major survival factor for limb undifferentiated progenitors and BMPs are pro-apoptotic factors expressed in the interdigits. In view of these results, we next analyzed whether cell death after trichostatin A treatment was associated with DNA damage as occurs physiologically in regressing interdigits [12]. We employed γH2AX immunolabeling as a precocious marker of DNA damage. As shown in Figure 5G,H, a region of intense positivity appeared in the interdigital cells around the bead after 8 h of treatment (*n* = 8).

## 4. Discussion

Embryonic development is a complex process in which the intense and continuous growth of the structures that build the body shares space and time with events of programmed cell death. It is now well known that histone variants and histone modifications establish chromatin states in the cells that play important roles in modulating gene activation and repression during the differentiation of tissues [48].

In this study, we characterized the distribution of histone epigenetic marks by immunohistochemistry and their association with DNA methylation in limb skeletal progenitors fated to die. We observed a close association between 5mC and trimethylation of histone H3 lysines. In contrast, acetylated histones H3K9ac and H4ac showed an inverse distribution compared to 5mC, consistent with the opposite transcriptional function of these epigenetic marks. Active genes possess promoters enriched for H3K9ac and H3K27ac, and the promoters of repressed genes are marked by H3K27me3, H3K9me3, and 5mC [49]. In the interdigital progenitors, replication-independent histone H3.3 was also very abundant. Consistent with the proposed role for this histone variant in transcriptionally active chromatin, our immunohistochemical study showed widespread distribution of this histone within the nucleoplasm in a pattern similar to the acetylated lysine 9 of histone H3 (H3K9ac) [50]. However, distinct from H3K9ac and H4ac, expression of H3.3 overlapped in some regions with zones positive for 5mC. This finding fits with the reported presence of H3.3 in the so-called bivalent gene promoters containing H3K4me3 and H3K27me3 that are dynamically activated or repressed during development [51,52,53,54]. Moreover, H3.3 enrichment is also observed in silent chromatin such as telomeres, where its presence correlates with the repression of telomeric RNA transcription [55]. 

During digit morphogenesis, the interdigital mesoderm is fated to undergo massive cell death [13]. Although, for decades, the cellular and molecular bases of these remodeling processes have been the focus of numerous investigations, some of these mechanisms are still controversial [8]. In previous studies, we described the sequence of degeneration as a succession of degenerative events in which DNA damage precedes caspase activation [12]. Based on these results, we proposed that the cause of death lies in the characteristics of cells that are going to die. It has been shown that interdigital cells retain the potential to form extra-digits but die when they are exposed to signals that promote cartilage differentiation unless they are previously stimulated by Smad 2–3 TGF-β signaling [56,57,58]. The pattern of modified histones observed in the interdigital tissue, together with the dying response preceded by DNA damage of interdigital cells subjected to HDAC inhibition, is consistent with the existence of a “chromatin remodeling checkpoint” that drives cells to apoptosis or senescence, via activation of p53 if the chromatin status is unappropriated to responds to transcription factors that initiate cell differentiation [59,60]. The partial inhibition of cell death and senescence by local application of FGFs together with the TSA bead supports this interpretation. Consistent with this observation, *Fgf8* was quickly downregulated by treatments with TSA. This observation supports a primary function of *Fgf8* loss of function in the activation of the degenerative process. The negative regulation of this gene may be explained by a precocious functional degeneration of the AER or by the activation of a repressor genes activated by TSA treatments. The moderate regulation of TSA-induced cell death by Noggin is consistent with the fact that only Bmp5 (out of the four BMPs expressed in the interdigits) was upregulated more than twofold in the 7 h time period covered by our transcriptional analysis. 

The mechanisms accounting for DNA breakage preceding the onset of interdigital cell death have been correlated with oxidative stress but, as proposed above, the histone epigenetic signature may contribute actively to this process [9,10,11]. Here, we observed that the area occupied by the acetylated histones remained constant in the course of interdigit remodeling. Acetylation of histones leads to partial decondensation of chromosomal domains that can impact the sensitivity of the DNA to damage [61]. This relaxed conformation of chromatin might favor the occurrence of DNA breaks that precede apoptosis during the regression of interdigital tissue. The observation of DNA damage positive for γH2AX immunolabeling a few hours after the interdigital application of trichostatin A supports this view.

From our observations, we cannot discard that the histone epigenetic profile of the interdigital cells may reflect a response to primary and mechanistically unrelated DNA damage. In other models, the presence of the histone variant H3.3 in the cell nucleus has been described as a signal of broken DNA, and in embryonic stem cells, elevated histone H3 lysine 9 acetylation (H3K9ac) contributes to muting the DNA damage response and increased radiosensitivity [62,63]. In the same way, histone H4 acetylation has been implicated in the recruitment of DNA damage repair proteins, especially during double-strand break repair [64,65].

In summary, according to our findings, interdigital progenitors fated to die exhibit chromatin enriched in acetylated histones. Furthermore, histone deacetylase inhibition, which promotes an open chromatin state, induces interdigital cell death and alters digit development. Together, these findings suggest that the epigenetic profile of the interdigital mesoderm contributes to the sensitivity to DNA damage that precedes apoptosis during tissue regression.

## Data Availability

Not applicable.
